# Mindfulness-based stress reduction in Parkinson’s disease: a systematic review

**DOI:** 10.1186/s12883-017-0876-4

**Published:** 2017-05-15

**Authors:** G. McLean, M. Lawrence, R. Simpson, S. W. Mercer

**Affiliations:** 1 General Practice and Primary Care, Institute of Health and Wellbeing, University of Glasgow, I Horselethill Road, Glasgow, Scotland G12 9LX UK; 2 Institute for Applied Health Research, School of Health and Life Sciences Glasgow Caledonian University, Glasgow, G4 0BA UK

**Keywords:** Mindfulness, Parkinson’s Disease, Systematic Review, Quality of Life, Depression, Brain

## Abstract

**Background:**

Mindfulness based stress reduction (MBSR) is increasingly being used to improve outcomes such as stress and depression in a range of long-term conditions (LTCs). While systematic reviews on MBSR have taken place for a number of conditions there remains limited information on its impact on individuals with Parkinson’s disease (PD).

**Methods:**

Medline, Central, Embase, Amed, CINAHAL were searched in March 2016. These databases were searched using a combination of MeSH subject headings where available and keywords in the title and abstracts. We also searched the reference lists of related reviews. Study quality was assessed based on questions from the Cochrane Collaboration risk of bias tool.

**Results:**

Two interventions and three papers with a total of 66 participants were included. The interventions were undertaken in Belgium (*n* = 27) and the USA (*n* = 39). One study reported significantly increased grey matter density (GMD) in the brains of the MBSR group compared to the usual care group. Significant improvements were reported in one study for a number of outcomes including PD outcomes, depression, mindfulness, and quality of life indicators. Only one intervention was of reasonable quality and both interventions failed to control for potential confounders in the analysis. Adverse events and reasons for drop-outs were not reported. There was also no reporting on the costs/benefits of the intervention or how they affected health service utilisation.

**Conclusion:**

This systematic review found limited and inconclusive evidence of the effectiveness of MBSR for PD patients. Both of the included interventions claimed positive effects for PD patients but significant outcomes were often contradicted by other results. Further trials with larger sample sizes, control groups and longer follow-ups are needed before the evidence for MBSR in PD can be conclusively judged.

## Background

Parkinson’s disease (PD) is a chronic neurodegenerative disease generally conceptualised as affecting motor function [1]. However, those with PD often face additional ‘non-motor symptoms’ (NMS), such as mental health comorbidities including depression and anxiety [2, 3]. These NMS have been associated with reduced quality of life in PD [4]. Depression and anxiety can also exacerbate the symptoms of PD [2, 5]. Therefore it is important to consider how therapies that can treat the symptoms of anxiety and depression, and increase resilience to stress may be beneficial to PD patients in dealing with their condition. People with PD have been found to be open to non-pharmacological treatment interventions [6, 7].

Mindfulness based stress reduction (MBSR) is an approach which is increasingly being used on people with long-term conditions. MBSR was first conceptualized and used by Jon Kabat-Zinn [8] and was developed as a complementary intervention in a hospital setting to serve as a referral service to physicians and other health care providers for patients with chronic pain or multiple chronic conditions who were not responding sufficiently to standard treatments [9]. MBSR and related approaches have also been associated with improvements for a range of conditions [10]. Reviews have demonstrated improvements in symptoms amongst people with cancer [11, 12] pain [13, 14] cardiovascular disease [15], stroke [16] and multiple sclerosis [17]. Improvements have included mental health outcomes, such as depression, stress and anxiety, as well as overall quality of life. Despite the potential benefits MBSR might have, no previous review has been undertaken to determine the effects of MBSR for the treatment of those with PD. Therefore, this systematic review aimed to determine the effectiveness of MBSR for patients suffering from PD.

## Methods

A registered protocol (PROSPERO 2016: CRD42016035304) guided the conduct of this review [18], which is reported in adherence to the Preferred Reporting Items for Systematic Reviews and Meta-analyses (PRISMA) Statement [19].

### Eligibility criteria

Inclusion criteria were based on the study design, participants, interventions and outcomes (SPIO), as described below. SPIO is an adaptation of the PICO (Population, Interventions, Comparison, Outcomes) framework [20] https://library.med.nyu.edu/library/instruction/handouts/pdf/picohandout.pdf. Trials which solely focus on, or incorporate a mindfulness intervention as defined by the author were selected. The population was adults diagnosed with PD. As with many other mind-body interventions, mindfulness as a therapeutic intervention is inherently varied and heterogeneous. Thus different forms, duration and frequency of mindfulness interventions were included. We included both Randomised Controlled Trials (RCTs) and non-randomised controlled trials which reported quantitative outcomes. Finally, we only considered studies published in peer-reviewed journals in English, as evidence suggests that limiting studies in this way does not introduce significant bias but is associated with considerable resource savings [21].

### Information sources and search strategy

The following databases were searched: CENTRAL (Cochrane), Medline, Embase, Amed, PsycINFO and CINAHL.

These databases were searched using a coMBSRnation of subject headings where available (such as MeSH) and key words relating to Mindfulness and PD. The search strategy was developed for use in Medline, and was amended for use in the other databases, using appropriate controlled vocabulary, Boolean operators and search symbols. The search was complemented by carrying out citation searches for included studies. We also scanned the reference lists of related reviews for potentially relevant papers.

### Study selection

Following de-duplication, bibliographic records (titles and abstracts) were downloaded into the Distiller software programme https://www.evidencepartners.com/products/distillersr-systematic-review-software/ Abstracts and full papers were screened against the inclusion criteria, by two reviewers working independently. Inter-reviewer disagreements were resolved by discussing whether the paper met the inclusion/exclusion criteria. If consensus between the reviewers was not possible, the decision was referred to a third party.

### Data extraction

We used online data collection forms using Distiller SR software. Two independent researchers extracted data on study details (country of origin, inclusion/exclusion criteria, number of participants), participant details (mean age, % male, ethnicity, socio-economic status, smoking status and comorbidities), description of the intervention, and outcomes.

### Assessment of methodological quality

We based assessment of quality on measures from the Cochrane collaboration tool for assessing bias [22] Quality was assessed in each of the included studies by the two researchers working independently. Methods of allocation concealment, randomisation procedure, dropout rate and whether there was evidence of selective outcome reporting were assessed by answering yes, no or unknown.

Meta-analysis was planned if sufficient homogeneous studies were available for statistical pooling. However, as only 2 studies (3 papers) were included and were of different designs with one reporting results compared to a control group and one having no control group, meta-analysis was not possible.

## Results

**Fig. 1:**
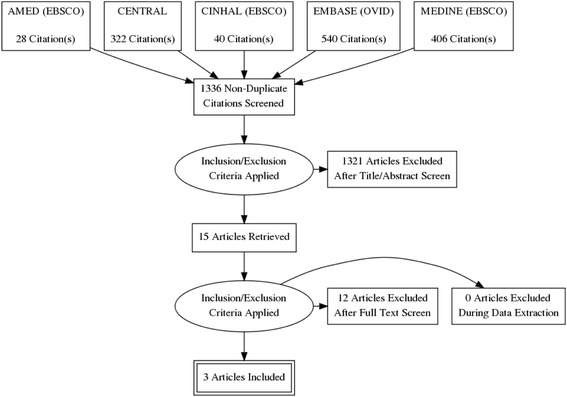
Flowchart for PD review

Our initial search identified 1336 papers. After title and abstract screening there were 15 papers for full paper review. Three papers from two interventions met our criteria and were included (see Fig. 1).

### Description of included studies

The two studies included a total of 66 participants, with 27 and 39 participants per study respectively (see Table 1). They took place in the USA [23] and Belgium [24, 25]. The studies differed considerably in the nature and delivery of the intervention and the outcome measures used. One study was a randomised control trial and one a non-randomised trial. The studies were similar in the age profile of the participants with mean age ranging from 61.8 [24, 25] to 65.6 [23]. Only one study reported the ethnicity of the participants and showed they were predominantly Caucasian [23]. Both studies had a majority of men ranging from 51.9% [24, 25] to 58.3% [23].

**Table 1:** Characteristics of included papers

Author (Year) Location	Population Numbers	Mean Age (SD)	Ethnicity	Main Outcomes assessed	Main results
Cash at al [22] 2016 USA	39 (21 men (53.8%) 18 women)	65.6 (7.6)	89.7% Caucasian	Apathy (Apathy Scale), depression (Patient Health Questionnaire-9); anxiety (Generalized Anxiety Disorder-7)	Mindfulness levels significantly increased for all participants from pre- too immediate- to post. A significant improvement was seen for self –reported symptoms of depression and self-reported language functioning. Also showed significant improvement on mental flexibility and complex attention tasks and reported significantly fewer emotional and cognitive symptoms associated with PD.
Pickut et al. [23] 2013 Belgium	27 (14 (51.9%) men 13 women)	61.8 (9.1)	N/I	MRI data sets of the brain were obtained at baseline and after 8 weeks’ follow-up. VBM analysis was performed using DARTEL from the SPM8 software. The resulting difference maps were statistically compared to examine grey matter density (GMD) differences.	Increased GMD was found in the MBSR compared to the UC group in the region of interest analysis in the right amygdala, and bilaterally in the hippocampus. Whole brain analysis showed increased GMD in the left and right caudate nucleus, the left occipital lobe at the lingual gyrus and cuneus, the left thalamus, and bilaterally in the temporo-parietal junction. In contrast, GMD differences were found in the UC group in the left anterior lobe and dentate nucleus of the cerebellum.
Pickut et al. [24] 2015 Belgium	27 (14 men 13 women)	61.8 (9.1)	N/I	Unified Parkinson’s Disease Rating Scale, Five Facet Mindfulness Questionnaire, PDQ-39, Beck Depression Inventory (BDI)	Significant changes after the MBSR were found including a 5.5 point decrease. On the UPDRS motor score, an increase of 0.79 points on Parkinson’s disease questionnaire (PDQ-39) pain item, and a 3.15 point increase in the Five Facet Mindfulness Questionnaire observe facet.

### Aims of the interventions

Cash et al. [23] was a pilot study of the feasibility and impact of an 8-week mindfulness-based group intervention on cognitive and emotional functioning for individuals with PD and their caregivers. The Picket et al. [24, 25] study produced two papers from the same intervention. The principal aim of one was to investigate structural changes on brain MRI using voxel based morphometry (VBM) [24] while the other investigated possible neurobehavioral and neuroplastic changes secondary to taking part in mindfulness training for individuals living with PD [25].

### Description of interventions

A summary of the key components of the interventions is given in Table 2. Both studies used MBSR interventions that were based the original MBSR program developed at the University of Massachusetts^.^ [23–25].

**Table 2:** Description of Interventions

Author (Year) Location	Intervention	Description
Cash at al [22] 2016 USA	MBSR	Participants were enrolled in 7–10 person groups that met weekly for 1.5-h-long sessions for 8 weeks at an interdisciplinary movement disorders clinic in a large, private room. The program included a half-day (4 h) silent retreat taking place approximately 1 week after the eighth session to encourage participants to deepen their practice by providing time for longer practices in a group setting.
Pickut et al. [23, 24] 2013 Belgium	MBSR	The intervention consisted of 2.5 h meetings on eight consecutive weeks. The intervention did not include an all day session. Audio recordings containing 45-min guided mindfulness exercises (meditation, body scan, mindful movement) were given with instructions for daily home practice corresponding to the course sequence.

Adaptations to the standard 8-week long program plan were varied across the studies. One study used sessions of shorter duration i.e. 1.5 h [23] compared to the traditional 2.5 h. The Cash et al. study [23] also used a later timing for the retreat at 1 week after the eighth session; the retreat session also had a shorter duration, at half a day, than the 1-day format generally used for MBSR programs. In contrast, Pickut et al. offered no full day retreat [24, 25].

All studies offered psycho-education, mindful meditations (sitting meditation, walking meditation) and gentle yoga. Cash et al. [23] encouraged participants to modify techniques as needed to meet their personal needs and limitations, such as sitting in a chair or lying down to optimize physical comfort and safety, or practicing chair yoga rather than floor-based exercises if unable to get up safely from the floor [23]. Both studies contained instructions for home study for the duration of the course [23–25].

### Quality appraisal

Details of the quality appraisal of the included studies can be found in Table 3. Only one intervention had a control group (usual care) and was therefore the only study considered to have a correct randomisation procedure [24, 25]. Allocation concealment was also clear in this intervention. One study had a dropout rate greater than 20% [23]. Both studies were adjudged not to have controlled appropriately for potential confounders in their analysis. In addition, study samples were relatively small in size, meaning that they were likely to be under-powered.

**Table 3:** Quality appraisal for included studies

Author (Year)	Appropriate Randomisation Technique	Allocation concealment	Dropout rate < 20%	Potential confounders properly accounted for	Were eligibility clear
Cash at al [22] 2016 USA	N/A	N/A	No	No	Yes
Pickut et al. [23, 24] 2013 Belgium	Yes	Yes	Yes	No	Yes

### Outcomes

#### PD measures

The impact on PD was measured by Pickut et al. [25] using the Unified Parkinson’s Disease Rating Scale (UPDRS) [26]. The investigator scoring, including UPDRS motor scoring was performed by a movement disorders specialist who was blinded as to the study allocation arm of the participant in question. Each question has a 5-point scale, where 0 means an absence of symptoms and 5 represents the most severe symptoms. They found significant change was obtained for the UPDRS motor III score (*F* = 4.39, *p* < .05), demonstrating motor changes for the MBSR group (pre-M = 27.43 versus post-M = 21.93), while no significant changes could be obtained for the UC group (pre-M = 27.92 versus post-M = 29) [25]. This revealed that after the MBSR a decrease of 5.5 (20.05%) points was found on the UPDRS motor score. Cash et al. [23] assessed non-motor function using the PD Non motor Symptoms Questionnaire [27] but found no significant differences.

### Depression

Two of the studies assessed the impact on depression using two different measures [23, 28]. Cash et al. [23] used the Patient Health Questionnaire-9 [29] and found that participants showed significant declines in their report of depression symptoms, F (1, 24) =6.31, *p* = .019, η2 = .21. However, Pickut et al. [25] used the Beck Depression Inventory (BDI) [30] and found no significant difference.

### Anxiety

Cash et al. [23] assessed the impact on anxiety using the Generalized Anxiety Disorder-7 [31] scale but found no significant differences.

### Quality of life measures

Both Cash et al. [23] and Pickut et al. [25] used the Parkinson’s disease questionnaire (PDQ-39) [32] which is a subjective questionnaire to assess quality of life in patients with PD. The questionnaire consists of 39 questions, which are divided into eight subscales (mobility, ADL, emotional well-being, stigma, social support, cognition, communication, and bodily discomfort). Each question has a range from 0 (no problem) to 100 (continuous problem/unable to do it). Cash et al. [23] reported that significant improvements were seen on the emotional functioning, F (1, 25) =10.34, *p* = .004, η2 = .29, and cognitive functioning, F (1, 25) =4.50, *p* = .044, η2 = .15, subscales. Pickut et al. [25] found that a marginally significant interaction effect (*F* = 3.50, *p* = .07) was obtained for the Parkinson’s disease questionnaire (PDQ-39) pain score, suggesting an increase of 0.79 (10.53%) points for the MBSR group and a decrease of 0.69 (8.63%) for the usual care group. However, no significant changes were found for the overall (PDQ-39) score for either study.

### Apathy

Cash et al. [23] used the Apathy scale [33] to measure changes in apathy but found no significant changes in levels.

### Mindfulness

Both Cash [23] and Pickut et al. [25] used Five Facet Mindfulness Questionnaire [34] (FFMQ) of 39 items; five domains of mindfulness “skills” are assessed: observing, describing, acting with awareness, non- judging of inner experience, and non-reactivity to inner experience. Items are rated on a 5-point Likert-type scale from “never or very rarely true” to “very often or always true.” The adding up of domain results in a total score gives an approximation of mindfulness “skills.” Cash et al. [23] found no significant changes. Picket et al. [25] found a significant interaction effect (*F* = 11.07, *p* < .01) was obtained for the FFMQ ‘observe’ facet of the scale indicating that for the MBSR group there was a significant increase after treatment (M = 27.29) in comparison with before treatment (M = 24.14). For the UC group, no difference was obtained between before (M = 23.69) and after treatment (M = 23.54). No significant effect was obtained for the other facets of the FFMQ.

### Brain magnetic resonance imaging (MRI)

Picket et al. [24] evaluated the neuroplastic effects of MBSR on the brain using anatomical MRI scans, assessing voxel-based morphometry (VBM). T1-weighted data sets were segmented into gray matter (GM), white matter (WM) and cerebrospinal fluid (CSF), using a unified tissue segmentation approach. For each individual patient, the registered baseline T1-weighted image was subtracted from the 8 weeks’ follow-up T1-weighted image to obtain images that represent changes over time for each patient. These subtracted images were then compared between both intervention groups using a T-test. MRI findings showed increased GMD in the MBSR group compared to the UC group overtime in the left and right hippocampi and a small region in the right amygdala. Whole brain analysis revealed a large cluster in the right caudate nucleus and a smaller cluster in the left caudate nucleus. Significant clusters were also found in the left occipital lobe at the lingual gyrus and cuneus, in the left thalamus and bilaterally in the tempero-parietal junction. However, increased GMD was found in the UC compared to the MBSR group in the anterior lobe and dentate nucleus of the left cerebellum.

## Discussion

This systematic review found only limited evidence that MBSRs can prove useful in PD patients due to the small number and nature of the studies that were eligible for inclusion. Both of the included interventions claimed positive effects for PD patients. These findings are also supported by a qualitative study of 12 people with PD which found that participation in a mindfulness-based intervention helped with making positive changes to coping responses and could benefit people with PD and was an acceptable form of group intervention. [35] However, where studies reported on similar outcomes, differences in results were found. Pickut et al. [25] found that the intervention improved PD outcomes in contrast to Cash et al. [23] who found no significant effects. Similarly, Pickut et al. [25] reported an improvement in mindfulness measured with the FFMQ but Cash et al. [23] found no significant changes with the same measure. Conversely, Cash et al. [23] reported an improvement in depression, but Pickut et al. [25] found no significant difference. Pickut et al. [24] found significantly increased grey matter density (GMD) in the MBSR group compared to the usual care group. However, the authors recognise that the effects of this signal increase need to be correlated with motor and non-motor symptoms of PD and until further studies verify this effect the evidence for beneficial effects for PD patients remains limited [24].

Methodological and quality differences between the included studies might explain some of the differences in results. Only one study [24, 25] used a control group (usual care) making it difficult to draw definite conclusions from the other study about the likley causal effect of the intervention and changes in outcomes found [28]. Both studies where small in size, and only one was of reasonable quality [24, 25]. Adverse events and reasons for drop-outs were not reported. This is of concern as the safety of MBSR may be particularly important for PD patient due to the nature of their condition [36]. There was also no reporting on the costs/benefit of the intervention. The studies that reported on ethnicity of the intervention subjects showed them to be largely Caucasian, while little information on the socio-economic status of the participants. Follow up periods were short, with both interventions recording results immediately post intervention, and thus beneficial impacts over a longer period of time could not be determined.

Since conducting our review a recent additional small exploratory RCT been published on an intervention that combined mindfulness with lifestyle education [37]. This found limited evidence of effectiveness of the intervention on the primary outcome of well-being (Parkinson’s Disease Questionnaire; PDQ-39). The author’s concluded that a larger study was required.

### Strength and limitations

This is the first review evaluating the effectiveness of MBSRs in PD patients and has been carried out rigorously. The limited number and design of the studies meant meta-analysis was not possible. The small number of studies also meant analysis of the possible effects of the different specific intervention components, such as shorter duration of the sessions and no full day retreat, was not possible.

## Conclusions

This systematic review found inconclusive evidence of the effectiveness that MBSRs have for PD patients. Further trials with larger sample size, control groups and longer follow-up are needed before the evidence for MBSRs in PD can be conclusively judged.
